# Home-based bimanual training based on motor learning principles in children with unilateral cerebral palsy and their parents (the COAD-study): rationale and protocols

**DOI:** 10.1186/s12887-018-1110-2

**Published:** 2018-04-18

**Authors:** Marlous Schnackers, Laura Beckers, Yvonne Janssen-Potten, Pauline Aarts, Eugène Rameckers, Jan van der Burg, Imelda de Groot, COAD Focus Group, Nicole Brouwers, Anke Defesche, Yvonne Geerdink, Bregtje Janssen, Marjon Kissels, Martijn Klem, Denise Martens, Judith Van Munster, Bianca Olive, Marleen Philippens, Lucianne Speth, Ingrid van den Tillaar, Rob Smeets, Sander Geurts, Bert Steenbergen

**Affiliations:** 1 Behavioural Science Institute, Radboud University, Nijmegen, the Netherlands; 2 Department of Rehabilitation Medicine, School for Public Health and Primary Care (CAPHRI), Maastricht University, Maastricht, the Netherlands; 3 Centre of Expertise in Rehabilitation and Audiology, Adelante, Hoensbroek, the Netherlands; 4 Department of Pediatric Rehabilitation, Sint Maartenskliniek, Nijmegen, the Netherlands; 5 Master of Specialized Physical Therapy, AVANS Plus, Breda, the Netherlands; 6 School of Pedagogical and Educational sciences, Radboud University, Nijmegen, the Netherlands; 7 Department of Rehabilitation, Donders Centre for Brain, Cognition and Behaviour, Radboud University Medical Centre, Nijmegen, the Netherlands; 8 Libra Rehabilitation and Audiology, Eindhoven, Weert the Netherlands; 9 School of Psychology, Australian Catholic University, Melbourne, Australia; 10 Centre for Disability and Development Research, Australian Catholic University, Melbourne, Vic Australia

**Keywords:** Cerebral palsy, Rehabilitation, Upper extremity, Home program, Task-specific training, Implicit motor learning, Explicit motor learning, Bimanual performance, Parental stress

## Abstract

**Background:**

Home-based training is considered an important intervention in rehabilitation of children with unilateral cerebral palsy. Despite consensus on the value of home-based upper limb training, no evidence-based best practice exists. Promoting compliance of children to adhere to an intensive program while keeping parental stress levels low is an important challenge when designing home-based training programs. Incorporating implicit motor learning principles emerges to be a promising method to resolve this challenge.

**Methods:**

Here we describe two protocols for home-based bimanual training programs, one based on implicit motor learning principles and one based on explicit motor learning principles, for children with unilateral spastic cerebral palsy aged 2 through 7 years. Children receive goal-oriented, task-specific bimanual training in their home environment from their parents for 3.5 h/week for 12 weeks according to an individualized program. Parents will be intensively coached by a multidisciplinary team, consisting of a pediatric therapist and remedial educationalist. Both programs consist of a preparation phase (goal setting, introductory meetings with coaching professionals, design of individualized program, instruction of parents, home visit) and home-based training phase (training, video-recordings, registrations, and telecoaching and home visits by the coaching team). The programs contrast with respect to the teaching strategy, i.e. how the parents support their child during training. In both programs parents provide their child with instructions and feedback that focus on the activity (i.e. task-oriented) or the result of the activity (i.e. result-oriented). However, in the explicit program parents are in addition instructed to give exact instructions and feedback on the motor performance of the bimanual activities, whereas in the implicit program the use of both hands and the appropriate motor performance of the activity are elicited via manipulation of the organization of the activities.

**Discussion:**

With the protocols described here, we aim to take a next step in the development of much needed evidence-based home-based training programs for children with unilateral cerebral palsy.

## Background

Cerebral palsy (CP) is a group of neurodevelopmental disorders of movement and posture [1, 2]. About one-third of children with CP experiences motor impairments predominantly affecting one side of the body, i.e. unilateral CP (uCP) [3], with impaired upper limb functioning as one of the most disabling symptoms [4]. Although the condition of CP is static, upper limb functioning is amendable to change, owing to the plasticity of the central nervous system [5]. Plasticity is the major entry point for the many rehabilitation programs that focus on improving upper limb functioning in these children [6]. This appears from studies on the effectiveness of centre-based rehabilitation programs for improving upper limb functioning such as goal-directed training (e.g. [7, 8]), constraint-induced movement therapy (CIMT) (e.g. [9, 10]), and hand-arm bimanual intensive training (HABIT) (e.g. [11, 12]). Studies examining these programs have shown that the key ingredients for effective treatment constitute high training intensity combined with meaningful, task-specific, bimanual training [13]. Crucially, in order to reach this high intensity, training needs to be motivating for the child, and accommodated to the child’s capabilities [11, 14, 15].

An important next step in rehabilitation practice is (the continuation of) training in the child’s daily life and home situation, such that empowerment of parents and independency from healthcare professionals of the parents and child are promoted. Furthermore, learning skills in the natural environment has been suggested to lead to better generalization of therapy effects [16]. In recent years, home-based training programs have been developed that have shown effectiveness [17]. However, at the same time these programs exemplified two important challenges that warrant further study in order for these programs to be feasible in the long term: 1) limit the therapy-related stress for the parents, and 2) promote compliance in children to adhere to an intensive program that involves repetitive practice [18, 19].

A critical remark on existing pediatric rehabilitation programs is their unspecified description and undifferentiated use of motor learning principles to train the children, i.e. explicit or implicit motor learning. In explicit motor learning conscious aspects of the motor learning process are targeted in particular, whereas in implicit motor learning especially non-conscious aspects of the motor learning process are targeted [20, 21]. Generally, a combination of implicit and explicit motor learning is used in therapy programs in clinical practice, but the main focus is on explicit principles [22]. In home-based training programs based on explicit motor learning principles, parents need to prompt the use of the affected side over and over again to maintain a high training intensity. This continuous prompting may impose an important stress factor upon parents [23], possibly reducing the motivation of both the parents and the child. A more feasible method for home-based training may be implicitly eliciting the (proper) use of the affected hand. In this way, the burden on parents to continuously prompt their child may be reduced. In addition, studies on basic motor learning in children with movement disorders have shown that implicit motor learning has positive effects on motivation [24, 25] and self-efficacy [26]. As a consequence, the increased motivation of the child to keep practicing may reduce parental stress levels, because they are less involved in continuously prompting their child.

Up until now, these promising advantages of implicit motor learning for home-based training have not been systematically studied. To enable this we have developed two home-based bimanual training programs. In this paper we present the protocols for two home-based training programs for young children with uCP, based on either implicit or explicit motor learning principles. A detailed description of the interventions is provided, in order to promote understanding of the content and to facilitate future research.

## Methods/Design

The description of the protocols follows the Template for Intervention Description and Replication (TIDieR) guide [27].

### General description

The two interventions described in this protocol are:

- a home-based bimanual training program based on implicit motor learning principles;
- a home-based bimanual training program based on explicit motor learning principles.

The overall aim of both home-based training programs is to improve the bimanual skills of the child whilst minimizing the increase of therapy-related parental stress.

The target population of the home-based training programs is children with unilateral spastic CP aged 2 through 7 years with Manual Ability Classification System (MACS) level I-III [28] and Gross Motor Function Classification System (GMFCS) level I-III [29]. A physiatrist will determine whether the intervention is applicable for a child and the parents. One or two caregivers (either the parents or significant others, for example a grandparent) will participate in the training, which will be determined in consultation with members of the rehabilitation team involved, before the start of the program. For reason of readability we will use the word ‘parents’ in this paper.

**Fig. 1:**
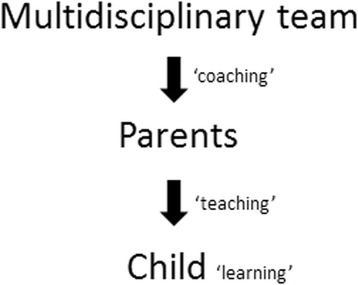
Triple action approach in home-based training programs

The execution of the home-based training programs follows a triple action approach (Fig. 1). Since previous studies have indicated that support by professionals promotes the feasibility of home-based training [30, 31], parents will be intensively *coached* by a multidisciplinary team, consisting of a pediatric therapist (occupational or physical therapist), and a remedial educationalist (or health care psychologist). As the focus of our home-based training programs is on the physical domain as well as the parent-child interaction and behavioral domain, parents will be coached by the therapist with regard to therapeutic content and implementation of the training in daily life, whereas the remedial educationalist will focus on the parent-child interaction and behavioral domain. In the programs, parents will *teach* the child new skills in the home environment. As a result, the child will *learn* new bimanual tasks in the needed context.

The actual home-based training is focused on improving the child’s bimanual performance of five personal rehabilitation goals (*goal-oriented*). To promote this process, a *task-specific* approach will be used, i.e. the activities will be consistent with the context of the particular goal. This task-specific approach is in agreement with the latest published version of the Dutch guidelines for treatment for children with spastic CP [32]. In these guidelines a task-specific intervention is defined as “the intervention is based on a task analysis aiming at practicing (sub-) activities that have been formulated in the goals” [32]. As proposed by Gordon [13] and based on the knowledge gained in centre-based programs, in both home-based training programs the same *high intensity* of bimanual training of meaningful task-specific activities is used.

Following the view on motor learning of Kleynen et al., the contrast between both programs is shaped by three elements: *instructions*, *feedback* and *organization* of the training [33].

Both home-based training programs consist of two phases, i.e. the preparation phase and the actual home-based training. Each phase comprises several intervention components (Table 1).

**Table 1:** Overview of phases and intervention components

Phase	Intervention component
Preparation of home-based training	Needs assessment and goal setting
	Introductory meetings with coaching professionals
	Design of individualized program
	Instruction of parents
	Home visit
Home-based training	Training
	Video-recordings
	Registrations
	Telecoaching and home visits by the therapist
	Telecoaching by the remedial educationalist

### Preparation of home-based training

The two-week preparation phase starts with setting goals for the home-based training. Next, introductory meetings between the parents and their child and the coaching professionals will take place. Thereafter the therapist will design the individualized program, while parents will be instructed concerning the home-based training. The last component of the preparation phase includes a home visit by the therapist. Each component will be elaborated on in the following paragraphs.

#### Needs assessment and goal setting

The parents will prioritize five important needs on the domain of bimanual activities using the Canadian Occupational Performance Measure (COPM) child-adapted version [34, 35]. The COPM is a semi-structured interview for parents, in which they identify and rank their child’s perceived problems in activities of self-care, play and/or leisure. The approach of this measure corresponds to the goal-oriented approach of the home-based training programs. The COPM has good psychometric properties [36, 37], which also apply to the child-adapted version [34]. The COPM will be administered over the phone by a therapist who is experienced in the COPM as well as in clinical practice in pediatric rehabilitation.

Later, the coaching therapist will transform the most important need into a SMART goal using video-recordings of the child’s performance of the corresponding activity, and will use Goal Attainment Scaling (GAS) to formulate predetermined criteria for the progress towards the rehabilitation goal to be scored [38].

#### Introductory meetings with coaching professionals

The parents and child will have a 60-min introductory meeting with the remedial educationalist and another 60-min meeting with the therapist at the rehabilitation centre to get acquainted and to discuss the professionals’ role in the coaching team. Furthermore, the remedial educationalist will examine aspects of the interaction and behavioral domain that are of interest for the home-based training, for instance the parent-child interaction and organization of family life (e.g. weekly schedule and division of family responsibilities between parents). The therapist and parents will discuss the family situation and home environment, e.g. objects available that are related to the individual rehabilitation goals. In addition, the therapist will observe and make video-recordings of the child’s performance of the activities corresponding to the needs.

#### Design of individualized program

To enable a task-specific training program, several steps are followed. The treatment goals, based on the COPM and the video-recordings the therapist made of the child’s performance of activities corresponding to the needs, as well as video-recordings of assessments, such as the Assisting Hand Assessment (AHA) [39], will provide the input. First, the coaching therapist will perform a task analysis based on stage 1 of the Perceive, Recall, Plan and Perform (PRPP) System of Task Analysis [40], by means of the video-recordings and an observation form. According to the PRPP system, each activity is separated into several logical successive steps of approximately equal size. As part of the task analysis, the therapist will evaluate the child’s performance of each single activity step regarding four error types:

- errors of omission (such as omitting hooking the zipper before pulling it up);
- errors of repetition (such as grasping and releasing the zipper several times before pulling up the zipper);
- errors of accuracy (such as being unable to hook the zipper, by missing the hooker);
- errors of timing (such as the time needed to close the zipper being too long).

Consequently, the therapist will design an individualized program. The order in which the treatment goals will be addressed is jointly decided upon by therapist and parents. The therapy activities focus on the important steps with the accompanying errors that emerged from the task analysis. Progress is realized by increasing the complexity of performance in each relevant step emerged from the task analysis. This can be accomplished by adjustments to the initial posture of the child in which the activity is performed, the choice of objects (e.g. size of the zipper) or the environment in which the activity is carried out (e.g. alone or accompanied by others).

#### Instruction of parents

Parents will be instructed how to apply the home-based training according to the specific home-based training program through instruction videos and a manual. The instruction of the parents will address four topics, i.e. 1) the content of the home-based training program, 2) the teaching approach, 3) the support by the therapist and remedial educationalist during the home-based training program, and 4) the use of a digital communication tool that will be used for safe communication and exchange of documents and videos between parents and the therapist and remedial educationalist during the home-based training.

#### Home visit

The preparation phase ends by a home visit by the coaching therapist lasting approximately 90 min. During this visit, the therapist will discuss the general outline of the designed individualized program with the parents, the therapist will examine the particular home situation with the parents, and parents have the opportunity to ask questions.

### Home-based training

The second phase consists of concurrently the actual training, video-recordings and registrations created and shared by parents, and coaching of parents by the therapist and remedial educationalist through telecoaching and home visits. The components will be discussed consecutively.

#### Training

Children will receive 3.5 h per week (on average 30 min per day) of bimanual training, for 12 weeks in total. To support the task-specific approach, the home-based training will be performed in meaningful situations, embedded in family routines. To accommodate this, the hours of training can be divided across the week in training sessions with a minimum duration of 10 min. The therapist and parents will select everyday objects or (therapeutic) toys that are appropriate for a specific rehabilitation goal. Use of objects and toys from the child’s home situation is preferred. The therapist will strive for activities and objects that are varied and in line with the possibilities of the child, to encourage children and parents and avoid frustration or boredom.

Despite the fact that at the start of the program an estimation is being made whether the goals are realistic for 12 weeks of training, it is possible that goals have already been reached before the end of the program. Should this situation arise, the activities are being repeated in order to maintain and automate the achieved progress. However, for the training to remain challenging and motivating, parents and children may indicate one or two new bimanual rehabilitation goals. These goals will be trained during the remaining weeks of the program, in addition to the repetition of the five initial goals.

An interruption of the program for 1 week is allowed, for example due to holidays or illness. In the event of a one-week break, this week will be compensated for at the end of the home-based training program. If because of circumstances beyond one’s control for more than 1 week cannot be trained, only 1 week will be made up for. The maximum duration of the home-based training program is therefore 13 weeks, namely 12 regular weeks and a maximum of one ‘catch-up week’.

#### Video-recordings

To enable the therapist and remedial educationalist to provide coaching that is tailored to the needs of the parents and child, parents will video-record a training session once a week. They will share the recordings with their coaching team through a digital communication tool. An extended and a short manual are available for both parents and the coaching team regarding the tool features needed for these interventions.

#### Registrations

Parents will register the training activities performed to obtain information about the actual intensity and content of the training. In both home-based training programs, parents register on a daily basis whether training activities were performed, and, if so, (1) how much time their child trained each rehabilitation goal, (2) the activities used to train each rehabilitation goal, and (3) particular details of that day (e.g. illness of the child). Daily registration time is expected to be approximately 5 min. Moreover, on a weekly basis, parents register the experiences of both the child and the parent(s) with the training that week by means of emoticons. Parents are expected to need 5 additional minutes per weekly registration. The registrations will provide the coaching team with the information needed for remote coaching.

The training activities will be registered using a digital form (Excel-sheet) including written instructions. The registration form will be partially pre-filled by the therapist in order to 1) reduce the time burden and 2) to increase the chance on comprehensiveness of the data. Parents will share the registration forms with their coaching team by the digital communication tool.

#### Telecoaching and home visits

At the start of each week, scheduled contact moments with the parents and their coaching therapist will take place. During these contact moments, a small standard evaluation will take place, in which parents have the opportunity to ask questions, address problems regarding the support of the child during training, and indicate whether they need additional coaching by the remedial educationalist. During this evaluation, the registrations and video-recording of the training will be discussed. Furthermore, the content of the program for the upcoming week is formulated. This weekly schedule ensures that adaptations to the original plan are possible. These contacts will be mainly over the phone, lasting approximately 30 min. Additionally, two times a home visit takes place, lasting approximately 60 min. The home visits will take place in week 5 and week 9 of the home-based training. During these home visits, the therapist will select objects and toys from the child’s home situation for the training in the upcoming weeks and may provide additional objects if necessary. If desired by the therapist, it is allowed to schedule one extra home visit as a replacement for a telephone contact moment.

The remedial educationalist will contact the parents over the phone in the third week of the home-based training program. During this 30-min contact, the remedial educationalist discusses with the parents the process of home-based training, the parent-child interaction and, if applicable, the sources of stress and how to cope with these. Hereafter, the remedial educationalist exchanges the findings with and gives advice to the therapist. In case parents have a need for extra support by the remedial educationalist or if the coaching therapist indicates the necessity, an additional contact moment with the remedial educationalist can be planned during the 12-week treatment period.

### Contrast between implicit program and explicit program

Both home-based training programs will contrast with respect to the teaching strategy, i.e. how the parents will support their child during training (Fig. 1). This is put into practice at the level of organization, instructions, and feedback [33] (Table 2).

**Table 2:** Contrast between implicit and explicit learning in home-based training programs

	Implicit	Explicit
Instruction and feedback	▪ Task-oriented ▪ Result-oriented	▪ Focused on motor performance ▪ Task-oriented ▪ Result-oriented
Organization	Eliciting	Prescribing

Parents participating in the *implicit* program will provide their child with instructions and feedback that solely focus on the activity itself (i.e. task-oriented) or the result of the activity (i.e. result-oriented) and that are aimed at motivating their child. A task-oriented instruction could for example be ‘please focus on closing the zipper’, whereas a result-oriented one would be ‘now try to close the zipper within ten seconds’. No information on how the child actually performs or should perform the activity is provided. That is, no information is given related to the movements needed to accomplish the activity. In this home-based training program, the use of both hands and the appropriate motor performance of the activity are elicited via manipulation of the organization of the activities. The organization of the activities comprises for instance the type of objects used (e.g. size of the zipper), the position of the child (e.g. sitting on the floor), and the setting (e.g. amount of distraction). Parents in this program will receive ideas from their coaching therapist, on activities and objects to elicit the proper bimanual performance by their child. Examples of corresponding instructions and feedback are also provided. During the home visits, the therapist will select a range of objects related to the rehabilitation goals that are available in the home environment and, if needed, additional objects.

Parents participating in the *explicit* program are instructed to give their child exact instructions and feedback on how to perform the bimanual activities, in addition to the instructions and feedback as described for the implicit program. In contrast to the implicit program, instructions in the explicit program are related to the movements needed to accomplish the activity, such as ‘hold the bottom of the jacket with your left hand while pulling the zipper up with your right hand’. Parents will receive specific and elaborate exercises with the corresponding instructions from the coaching therapist. Only specific objects necessary for the execution of the exercises will be selected in the home environment, and, if needed, provided.

To enforce this contrast, the task analyses in the explicit program will be complemented by a movement analysis for each step in which a performance error is detected. This movement analysis will focus on the child’s current performance (posture and movements) and the performance needed to complete the activity successfully. Based on this information, therapists operating in the explicit group can provide the parents with the instructions and feedback related to the appropriate motor performance.

### Course for the therapists and remedial educationalists

In order to coach the parents according to the specific home-based training program, therapists and remedial educationalists will be instructed by members of the research team. The course will take place prior to the start of inclusion of participants in the programs.

Therapists will be instructed during a one-day course by a physical therapist and an occupational therapist who have extensive experience with clinical practice, research and education in the field of pediatric rehabilitation. To prevent contamination, the instruction of the therapists will be provided for each home-based training program separately. During the course, therapists will be instructed how to perform a task analysis based on stage 1 of the PRPP System of Task Analysis [40], how to design the individualized training programs, and how to coach the parents during the intervention period. In addition, the use of a digital communication tool that can be used for the communication and exchange of documents and videos with parents will be addressed.

The course for remedial educationalists will last half a day. A remedial educationalist of the research team who has extensive experience with clinical practice, research and education in the field of pediatric rehabilitation will instruct the remedial educationalists operating in the home-based programs on how the parents should be coached during the intervention period, and on how to use the digital communication tool for the communication and exchange of documents with parents.

One year after the first instruction, a refresher course will be organized for all practitioners. For questions, therapists can contact the research team at any time.

The home-based training programs are elaborated in manuals. In these manuals, instructions and checklists are provided for all parts of the home-based training programs.

### Study organization

The interventions described in this protocol are part of the ‘Co-creation at hand: the road to independence’ (COAD) study. The COAD-study is a collaboration between Maastricht University, Adelante, Radboud University, Sint Maartenskliniek, and Radboud University Medical Center. The home-based training programs and study were designed in consultation with a focus group consisting of the director of the Dutch association of people with physical disabilities, parents of children with CP, an adolescent with CP, as well as rehabilitation physicians, occupational and physical therapists, and a remedial educationalist experienced with rehabilitation of children with CP.

A process evaluation will be performed, with which we aim to systematically evaluate the processes and factors that influence implementation and effects of our home-based training programs. The methods of the process evaluation are described in a parallel paper (Beckers L, van der Burg J, Janssen-Potten Y, Rameckers E, Aarts P, Smeets R: Process evaluation of two home-based bimanual training programs in children with unilateral cerebral palsy (the COAD-study): protocol for a mixed methods study, submitted). In addition, a case series study will be executed to investigate the effects of the programs on the level of the child and the impact on the parents.

## Discussion

In this paper we present two protocols for intensive home-based bimanual training in young children with uCP and their parents. Home-based training is considered an important intervention for rehabilitation care of children with CP, now and in the future. In home-based training, children learn new skills in their natural environment. This has been suggested to lead to better generalization of therapy effects [16]. For parents of children with CP, home-based training offers the possibility to become more engaged in the therapy of their child. This is in line with the framework of ‘family-centred care’ in which care is built on partnerships between parents and professionals [41]. This framework is regarded as the gold standard in therapy for children with CP [41, 42] and is claimed to enhance health outcomes [41]. Moreover, home-based training is relevant from a societal perspective, as it may lead to a reduction of healthcare costs [43].

Despite consensus on the importance of home-based upper limb training for all those involved [18], no evidence-based best practice exists yet. We aim to take the next step in the development of effective home-based upper limb training programs for children with uCP that are feasible in daily life situations. In order to provide intensive training in the home situation, it is essential that children are motivated and parents experience little stress. Previous studies, however, showed an increase in parental stress and a reduction of therapy compliance over time [18, 19, 44]. Incorporation of implicit motor learning principles seems to be a promising method to prevent or reduce these adverse effects. That is, implicit motor learning is expected to lead to improved motivation for training in children [24, 25], and to a reduced burden on parents due to less need for prompting their child to use the affected arm and hand properly. Moreover, we strive to resolve the challenges encountered in previous studies by coaching the parents not only with respect to bimanual performance of their child, but also with regard to parent-child interaction during practicing. Given the nature of the challenges observed in previous studies, this additional coaching by the remedial educationalists may be crucial. Because of their specific expertise on parent-child interaction and behavioral domain, the remedial educationalists are expected to be valuable in supporting the parents in their new role and in advising them on how to support and motivate their child. This will be important to empower the parents in their new role to facilitate an intensive training program.

Training intensity has been a crucial consideration regarding the feasibility of our home-based training programs. Studies on centre-based programs have shown that a high training intensity, i.e. 60–90 h in total, is essential for the improvement of upper limb functioning in children with CP [13]. However, a crucial difference between centre-based and home-based programs is the role of the parents in their child’s therapy. In contrast to centre-based programs, parents have a pivotal role in home-based programs as the facilitator(s) of their child’s training. However, their role has to be fulfilled in combination with their roles as parents and wage earners. A previous study on home-based training has shown that if training hours are not standardized, children with CP and their parents train, on average, 1–1.5 h per week [16]. Combining these findings, we have strived to adopt a middle course resulting in a training intensity of 3.5 h per week for 12 weeks (42 h in total). Through this training intensity, we aim for a level of parental involvement that is feasible for a heterogeneous group of parents to be able to implement the home-based training programs in a larger population.

## Abbreviations

AHA: Assisting hand assessment
CIMT: Constraint-induced movement therapy
COAD: ‘Co-creation at hand: the road to independence’
COPM: Canadian occupational performance measure
CP: Cerebral palsy
GMFCS: Gross motor function classification system
HABIT: Hand-arm bimanual intensive training
MACS: Manual ability classification system
PRPP System of Task Analysis: Perceive, recall, plan and perform system of task analysis
TIDieR: Template for intervention description and replication
uCP: Unilateral cerebral palsy
